# Meningioma brain tumor detection and classification using hybrid CNN method and RIDGELET transform

**DOI:** 10.1038/s41598-023-41576-6

**Published:** 2023-09-04

**Authors:** B. V. Prakash, A. Rajiv Kannan, N. Santhiyakumari, S. Kumarganesh, D. Siva Sundhara Raja, J. Jasmine Hephzipah, K. MartinSagayam, Marc Pomplun, Hien Dang

**Affiliations:** 1 Faculty of Information Technology, Government College of Engineering, Erode, Tamil Nadu India; 2 Faculty of Computer Science and Engineering, K.S.R College of Engineering, Namakkal, India; 3 Department of ECE, Knowledge Institute of Technology, Salem, Tamil Nadu India; 4 Faculty of Electronics and Communication Engineering, SACS MAVMM Engineering College, Madurai, Tamil Nadu India; 5 Faculty of Electronics and Communication Engineering, R.M.K. Engineering College, Kavaraipettai, Tamil Nadu India; 6 Department of ECE, Karunya Institute of Technology and Sciences, Coimbatore, India; 7 Department of Computer Science, University of Massachusetts Boston, Boston, MA USA; 8 Department of Mathematics and Computer Science, Molloy University, Rockville Centre, NY USA; 9 Faculty of Computer Science and Engineering, Thuyloi University, Hanoi, Vietnam

**Keywords:** Computational biology and bioinformatics, Mathematics and computing

## Abstract

The detection of meningioma tumors is the most crucial task compared with other tumors because of their lower pixel intensity. Modern medical platforms require a fully automated system for meningioma detection. Hence, this study proposes a novel and highly efficient hybrid Convolutional neural network (HCNN) classifier to distinguish meningioma brain images from non-meningioma brain images. The HCNN classification technique consists of the Ridgelet transform, feature computations, classifier module, and segmentation algorithm. Pixel stability during the decomposition process was improved by the Ridgelet transform, and the features were computed from the coefficient of the Ridgelet. These features were classified using the HCNN classification approach, and tumor pixels were detected using the segmentation algorithm. The experimental results were analyzed for meningioma tumor images by applying the proposed method to the BRATS 2019 and Nanfang dataset. The proposed HCNN-based meningioma detection system achieved 99.31% sensitivity, 99.37% specificity, and 99.24% segmentation accuracy for the BRATS 2019 dataset. The proposed HCNN technique achieved99.35% sensitivity, 99.22% specificity, and 99.04% segmentation accuracy on brain Magnetic Resonance Imaging (MRI) in the Nanfang dataset. The proposed system obtains 99.81% classification accuracy, 99.2% sensitivity, 99.7% specificity and 99.8% segmentation accuracy on BRATS 2022 dataset. The experimental results of the proposed HCNN algorithm were compared with those of the state-of-the-art meningioma detection algorithms in this study.

Meningioma, glioblastoma, and the hypothalamus are distinct forms of brain tumors. Meningiomas are often non-cancerous tumors that grow in thin walls and typically encircle the brain. Brain tumors are among the disorders that directly endanger human lives. Precise knowledge of the brain tumor phases is crucial for disease prevention and treatment. This study aimed to determine whether the brain is healthy or abnormal. In contrast, it specifies the type of tumor if an anomaly is detected. With the advent of machine learning, MRI image processing has become essential for rapid and accurate identification of brain tumors. Currently, there are different types of meningioma tumors at present. However, Clival, Convexity, and Suprasellar meningiomas are the main types of meningioma. The first two types of meningioma tumors were identified as mild, and the third type was identified as severe. In the United States, Approximately 38% of patients are affected by meningioma brain tumors. It mostly affects older people, as stated in the World Health Organization (WHO) report 2021 (https://www.cancer.net/cancer-types/meningioma/statistics). Figure 1 shows an MRI imageof a brain meningioma^1^.

**Figure 1:**
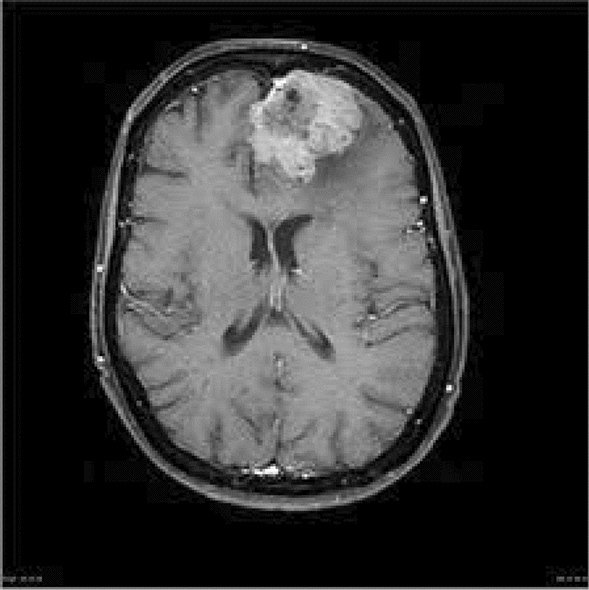
Meningioma brain MRI^1^.

The diagnosis of a patient is dependent on the manual assessment of the patient by a doctor as well as the test findings of the patient. In addition to the greater possibility that a doctor may make an incorrect diagnosis owing to the lack of automated technologies that can assist with diagnosis and the restricted number of physicians available, there is also a longer wait period for patients to be seen. Instead of investing time with the patient, doctors were required to manually evaluate test findings and photographs. This requires a valuable appointment time. It is vital to have improved medical technology in terms of automatic learning to increase the efficiency of doctors, which will, in turn, reduce the amount of time patients spend in hospitals and the amount of time it takes for patients to recover. This study aimed to create automated methods that will assist physicians in identification to reduce the amount of time that patients are required to wait for treatment and to avoid incorrect diagnoses. In particular, the automation of this process is accomplished by this study via the categorizing different forms of brain tumors based on photographs of the patient’s brain. When analyzing images, a physician must look at several image slices to identify potential health problems, which require months away from more difficult diagnoses. These constraints were eliminated because of the implementation of deep learning algorithms in the tumor picture identification process. In this study, the structure of deep learning was altered with respect to the centric technique. The centric method uses a shorter computing time period for the categorization of brain pictures compared to current deep learning methods.

## Literature survey

Although machine learning has applications in a wide variety of sectors, the vast majority of research has concentrated on its use in agriculture^2^ and healthcare to detect, predict, and classify illnesses^3,4^. The study of breast cancer takes precedence over research in other areas of medicine. The detection and segmentation of lung and colon cancers, the detection and segmentation of lung and brain tumors, and the categorization and diagnosis of respiratory and brain tumors have been presented^5^. The diagnostic procedure, which also involves excision and clinical investigation using a variety of cellular (histological) testing methods, is considered the diagnostic gold standard for brain tumors. Unfortunately, identification using biopsy is intrusive, which may lead to bleeding and possibly damage, which can lead to a loss of function, as stated by Roberts et al.^6^.

Consequently, noninvasive diagnosis of brain tumors using electromagnetic resonance imaging is the backbone of contemporary neuroimaging. This allows physicians to evaluate the morphological, molecular, metabolic, and functional characteristics of brain tumors, as stated by Roberts et al.^6^. White Matter (WM), gray matter (GM), and spinal fluid are the three components that may be observed in a usual operational MRI scan of a healthy brain, as stated in Rosenbloomet al.^7^. When performing a functional MRI scan, the degree to which these tissues vary is mostly determined by the amount of water they contain. Myelinated axons make up a snowy substance that seems to be composed of 70 percent water and is responsible for connecting the cerebral cortex to other parts of the brain. In addition, it acts as a conduit for the dissemination of data among nerve cells, and links the correct and leftward sides of the brain. Glial and neuronal cells, which are responsible for controlling brain activity, as well as cores, are found profoundly inside the brain substance and are composed of 80 percent water.

Fuzzy C Means (FCM) was used to determine the grade value of tumors as stated by Tiwari et al.^8^. A fuzzy cognitive mapping soft-computing system was used to represent and simulate the professional data. This method was used in this study for classification and precise grading. Despite this fact comprises two stages: the first stage involves charting a wanderer web based on wavelet information intended for the purpose of feature extraction, and the additional stage involves organization by means of a probabilistic neural network, which is applied to the features that have been extracted^9^. For the purpose of classification, adopted a backpropagation neural network approach^10^. Wavelet decomposition was used for feature extraction, and principal component analysis was used for the selection of features to incorporate decreased data and obtain improved outcomes. The findings of this approach were 100% accurate and required 0.0451 s to complete. However, the Support Vector Machine (SVM) classification approach was used by another author^11^. This Internet-based brain tumor library provides a source for an MRI image database of 140 brain tumors. When analyzing the data for tumor detection, a large dataset was employed, which resulted in a significantly enhanced quality. Shape, intensity, and texture are the three criteria used in the feature extraction process. Bal et al.^12^ used computerized MRI segmentation with the FCM clustering approach to create segments. A total of 820 photographs were retrieved. A MATLAB toolbox implementation of an SVM classifier was used for classification. This implementation resulted in an increase in accuracy of 97.95%. Radiologists can make diagnostic decisions based on the information provided by the system. Some machine learning and deep learning techniques have also been used by Bruntha et al.^13^ and Andrushia et al.^14^ for image classification to aid in the early detection of diseases.

Kumar et al.^15^ enhanced the accuracy by utilizing an image thickening and background thinning method to extract the performance calculation measurements. According to Elayaraja et al.^16^, a Genetic Algorithm (GA)—based convolutional neural network (CNN) classification process for segmenting particularized segments was developed, achieving 90.37% Se, 98.9% Sp, and 95.21% Ac. Thiyaneswaran et al.^17^ used k-means clustering in skin images to detect and segment cancerous regions. The authors achieved an average accuracy of 90.0% for open-access datasets. Kumarganesh et al.^18^ suggested using an adaptive fuzzy inference system (ANFIS) classifier system to detect tumors in basic images. They had a classification accuracy of 96.6%. Thiyaneswaran et al.^19^ calculated that AlexNet with an ADAM solver achieved a system accuracy of 98.21%. Kumarganesh et al.^20^ proposed an ANFIS classifier method to classify tumors from foundation pictures. They attained a sensitivity of 93.07%, specificity of 98.79%, and cancer segmentation accuracy of 97.63%.

The novelty of this paper is stated below.

- The novel CNN architecture is proposed by modifying the conventional CNN architecture.
- A novel meningioma brain tumor segmentation algorithm was proposed for segmenting tumor pixels more accurately.

## Proposed methods

**Figure 2:**
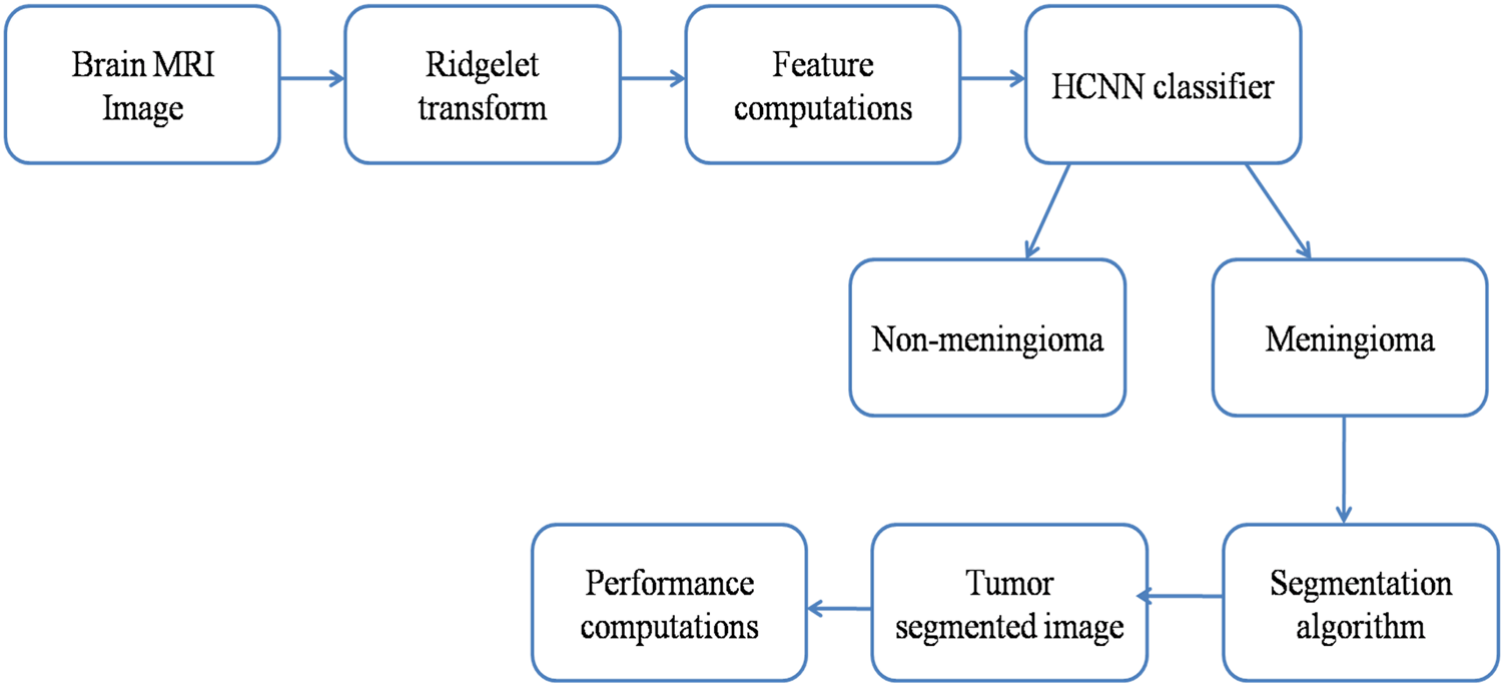
HCNN classifier-based brain image classification system.

In the existing meningioma brain tumor detection process, a conventional CNN architecture is used for brain image classification. The conventional CNN method is structured using a large number of convolutional, pooling, and dense layers, which reduces the classification rate and increases classification time. These drawbacks were overcome by proposing a novel and highly efficient HCNN classifier to detect and classify meningioma brain images from non-meningioma brain images. The HCNN classification technique consists of the Ridgelet transform, feature computations, HCNN classifier, and segmentation algorithm. Figure 2 shows the proposed HCNN classifier for the classification of brain images.

### Ridgelet transform

Because wavelets have been used for image denoising and decomposition over the past two decades, the pixel stability during the decomposition process is low. In addition, the singular directivity of the wavelet transform is poor. To reduce the error rate during the decomposition process, the singular directivity and pixel stability should be as high as possible.

Therefore, the Ridgelet transform was used in this study instead of the wavelet transform to decompose the source brain image into a number of subbands.

The Ridgelet transform is defined in the following equation.

1 $$R\left(i,j\right)=\left\{\left(i,j\right):j={k}_{i}+l \left(mod p\right)\right\};$$

where, ${k}_{i}$ is the radon projection factor and $p$ is the histogram counts.

The Ridgelet approach decompose the image into Ridgelet coefficients $R\left(i,j\right).$

### Feature computations

From the Ridgelet coefficients $R\left(i,j\right)$, the features related to the pixel intensity in the brain image are computed. In this study, Pixel Intensity Feature (PIF), Pixel Variation Feature (PVF), Pixel Mean Feature (PMF), first-order Intensity Feature (FIF), and second-order Intensity Feature (SIF) were used.

2 $$Pixel\; Intensity\; Feature \left(PIF\right)=\frac{\sum_{i=1}^{C1}\sum_{j=1}^{C2}{R(i,j)}^{2}}{{i}^{2}\times {j}^{2}}$$

where $R(i,j)$ is the coefficient of the ridgelet-transformed image and C1 and C2 represent the number of rows and columns in $R(i,j)$.

3 $$Pixel \;Variation\; Feature \left(PVF\right)=\frac{\sum_{i=1}^{C1}\sum_{j=1}^{C2}{((R\left(i,j\right)-i)}^{2}+\sum_{i=1}^{C1}\sum_{j=1}^{C2}{((R\left(i,j\right)-j)}^{2}}{i\times j}$$

4 $$Pixel\; Mean \;Feature \left(PMF\right)=\frac{\sum_{i=1}^{C1}\sum_{j=1}^{C2}R(i,j)}{i\times j}$$

5 $$First \;order\; Intensity \;Feature \left(FIF\right)=\frac{\sum_{i=1}^{C1}\sum_{j=1}^{C2}R\left(i,j\right)\times i\times j}{\left(i+1\right)(j+1)}$$

6 $$Second\; order \;Intensity\; Feature \left(SIF\right)=\frac{\sum_{i=1}^{C1}\sum_{j=1}^{C2}R\left(i,j\right)\times {i}^{2}*{j}^{2}}{{\left(i+1\right)}^{2}{(j+1)}^{2}}$$

These computed pixel intensity features are fed into the proposed HCNN classifier for an effective classification process.

### Proposed HCNN classifier

**Figure 3:**
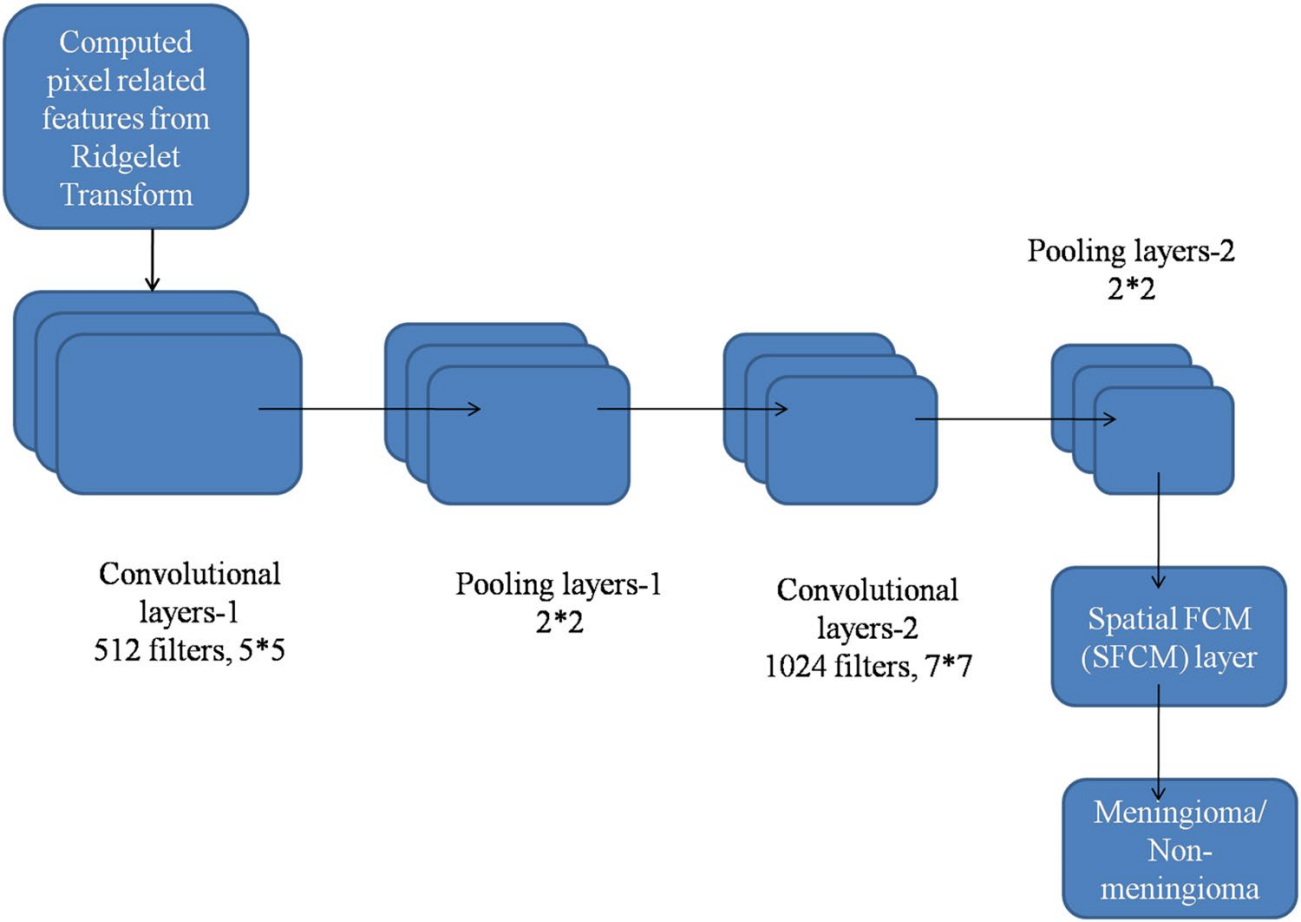
Proposed HCNN classifier using SFCM layer pattern.

Classifiers play an important role in brain-image classification. In the existing meningioma image classification process, the conventional CNN architecture is used to perform meningioma and non-meningioma image classification processes. The CNN architecture, which is used in existing methods, receives brain images as an input pattern and produces output using the internal features that are generated through the internal layers in the conventional CNN architecture. Although this increases the classification rate of the HCNN approach, there is no optimal meningioma image classification. Therefore, the conventional CNN architecture was modified into an HCNN classification architecture that combines deep learning and machine learning modules, as depicted in Fig. 3.

The proposed HCNN architecture for meningioma and non-meningioma image classification system consists of two convolutional layers (Convolutional layer-1 and Convolutional layer-2) and two pooling layers (Pooling layer-1 and Pooling layer-2) and a Spatial Fuzzy C Means (SFCM) layer at the output. Convolutional layer 1 in the proposed HCNN architecture consisted of 512 filters with a 5 × 5 stride function. The Convolutional layer21 in the proposed HCNN architecture consisted of 1024 filters with a 7 × 7 stride function. The pooling layer-1 was placed between these two convolutional layers to reduce the output size of convolutional layer 1. The pooling layer-2 was placed at the output of Convolutional layer-2 to reduce the output size from convolutional layer 2. The pooling layer output responses were then transferred to the SFCM layer to produce the classification results (either meningioma or non-meningioma).

**Figure 4:**
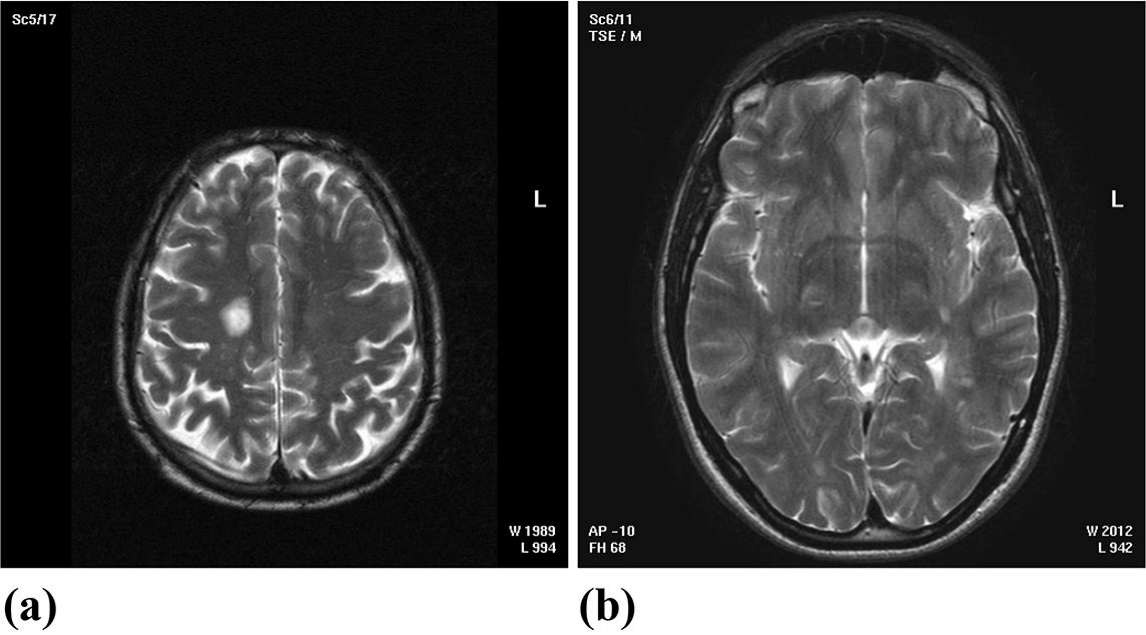
(**a**) Meningioma images (**b**) Non-meningioma images^1^.

Figure 4a,b illustrate the images classified using the HCNN classifier-based meningioma detection system.

### Segmentation

After the classification process was completed, the tumor pixels in the meningioma images were segmented using the probability-based algorithm proposed in this study. The steps of the proposed morphological segmentation approach are as follows.

## Step 1:

Apply morphological open on meningioma brain image $M (i,j)$ using the following equation.

7 $${M}_{o}=open (M\left(i,j\right))$$

## Step 2:

Apply morphological close on meningioma brain image $M (i,j)$ using the following equation.

8 $${M}_{c}=close (M\left(i,j\right))$$

## Step 3:

Find probability density functions of opened and closed image using the following equation.

9 $$p1=pdf({M}_{o})$$

10 $$p2=pdf({M}_{c})$$

## Step 4:

Find the average value of the computed probability density functions of the open and closed images using the following equation:

11 $${A}_{t}=\frac{1}{2}(p1+p2)$$

## Step 5:

Compute probable open and close image using the following equations.

12 $${P}_{open}=\frac{{M}_{O}}{{A}_{t}}$$

13 $${P}_{close}=\frac{{M}_{c}}{{A}_{t}}$$

## Step 6:

Compute difference image between probable open and close image using the following equation.

14 $${M}_{diff=| {P}_{open}-{P}_{close} |}$$

### Results and discussions

The MATLAB R2020 version was used to simulate the HCNN method in this study, and the simulation dataset was constructed by obtaining images from the BRATS 2019^1^ and Nanfang datasets^21^.

The BRATS 2019 dataset holds 350 numbers of meningioma images and 360 number of non-meningioma images. Among these images, 175 non-meningioma and 180 meningioma images are accessed from the dataset and being used for training the proposed system in this work. Moreover, another 175 non-meningioma and 180 meningioma images are accessed from the dataset and being used for testing the proposed system in this work. The size of the images in BRATS 2019 is about 240 × 240 and the images are quantized to 8 bit pixel resolution.

The Nanfang University dataset holds totally 600 non-meningioma images and 512 meningioma images for evaluating the proposed system. Among these images, 300 non-meningioma and 256 meningioma brain MRI images are accessed from the dataset and being used for training the proposed system in this work. Moreover, another 300 non-meningioma and 256 meningioma brain MRI images are accessed from the dataset and being used for testing the proposed system in this work. The size of the images in Nanfang university is about 512 × 512 and the images are quantized to 8 bit pixel resolution.

Table 1 shows the analysis of classification accuracy based on different classification algorithms on the BRATS 2019 dataset. The methodology for the meningioma brain tumor detection system stated in this paper achieved **99.7** a classification accuracy of 99.7%for the BRATS 2019 dataset.

**Table 1:** Analysis of classification accuracy based on different classification algorithms on BRATS 2019 dataset.

Classifiers	Classification accuracy (%)
Proposed HCNN classifier (in this paper)	**99.7**
ANFIS classifier	97.2
SVM	95.9
NN	94.3
Adaboost classifier	94.8
Fuzzy C Means classifier	93.9

Significant values are in bold.

The HCNN method was tested by replacing the proposed HCNN classifier with conventional machine learning classification algorithms to verify the effectiveness of the proposed meningioma detection process on the Nanfang dataset brain images.

The tumor segmentation technique using Adaptive Neuro Fuzzy Inference System (ANFIS) classifier obtains 97.2% of classification accuracy, using SVM classifier obtains 95.9% of classification accuracy, using Neural Networks (NN) classifier obtains 94.3% of classification accuracy, using Adaboost classifier obtains 94.8% of classification accuracy and using Fuzzy C Means classifier obtains 93.9% of classification accuracy, on the Nanfang dataset brain images.

Furthermore, the Nanfang dataset was used in this study to verify the effectiveness of the HCNN-based meningioma classification technique.

The proposed system received the test brain MRI image from the testing dataset, and the testing function of the proposed algorithm was executed against the trained patterns. The HCNN methodology proposed in this study achieved **99.36**% classification accuracy on the Nanfang dataset brain images.

Table 2 shows the classification accuracy analysis based on the feature combinations (HCNN classification results) for the Nanfang dataset.

**Table 2:** Analysis of classification accuracy based on different classification algorithms on Nanfang dataset.

Classifiers	Classification accuracy (%)
Proposed HCNN classifier (in this paper)	**99.36**
ANFIS classifier	95.29
SVM	93.98
NN	92.19
Adaboost classifier	90.76
Fuzzy C Means classifier	91.76

Significant values are in bold.

The HCNN-based meningioma classification technique was tested by replacing the proposed HCNN classifier with conventional machine learning classification algorithms in this study on the Nanfang dataset brain images.

The HCNN-based meningioma classification technique obtains 95.29% classification accuracy, the SVM classifier obtains 93.98% classification accuracy, the NN classifier obtains 92.19% classification accuracy, the Adaboost classifier obtains 90.76% classification accuracy, and the Fuzzy C Means classifier obtains 91.76% classification accuracy on the Nanfang dataset brain images.

Further, the proposed method is applied and tested on the recent BRATS 2022 dataset and the experimental results of this dataset are compared with the existing datasets BRATS 2019 and Nanfang in this paper. Table 3 shows the classification accuracy comparisons with respect to different datasets used in this paper.

**Table 3:** Classification accuracy comparisons with respect to different datasets used in this paper.

Datasets	Classification accuracy (%)
BRATS 2022	99.81
BRATS 2019	**99.7**
Nanfang university dataset	**99.36**

Significant values are in bold.

Transforms are an important processing module in the meningionma image detection system; hence, the proposed HCNN-based classification technique was analyzed based on different transforms. In this study, different transforms were applied to decompose brain images, and their performances were compared in terms of classification accuracy.

Table 4 presents the analysis of classification accuracy based on different transforms of the BRATS 2019 dataset. The proposed HCNN based classification technique using Ridgelet transform attained 99.7% of classificaytion accuracy, where the proposed tumor segmentation technique using Gabor transform attained 93.8% of classificaytion accuracy, using Discrete Wavelet Transform (DWT) attained 92.1% of classificaytion accuracy and using Non-Sub sampled Contourlet Transform (NSCT) attained 94.8% of classificaytion accuracy.

**Table 4:** Analysis of classification accuracy based on different transforms on BRATS 2019 dataset.

Transforms	Classification accuracy (%)
Ridgelet	**99.7**
Gabor transform^22^	93.8
DWT^23^	92.1
NSCT^24^	94.8

Significant values are in bold.

Table 5 illustrates the impact of different transforms on Nanfang dataset images.

**Table 5:** Analysis of classification accuracy based on different transforms on Nanfang dataset.

Transforms	Classification accuracy (%)
Ridgelet	**99.36**
Gabor transform	92.1
DWT	90.7
NSCT	91.5

Significant values are in bold.

Table 6 lists the classification accuracies of the BRATS 2019 dataset. The proposed meningioma detection system obtains a classification accuracy of 47.9% using PIF, 49.0% using PVF, 51.9% using PMF, 50.6% using FIF, 55.3% using SIF, 71.9% using PIF and PVF, 74.3% using PIF and PMF, 75.1% using PIF and FIF, 93.8% using PIF + PVF + PMF + FIF, and 93.8% using PIF + PVF + PMF + FIF + SIF features.

**Table 6:** Analysis of classification accuracy based on different features on BRATS 2019 dataset.

Features	Classification accuracy (%)
PIF	**47.9**
PVF	49.0
PMF	51.9
FIF	50.6
SIF	55.3
PIF + PVF	71.9
PIF + PMF	74.3
PIF + FIF	75.1
PIF + PVF + PMF + FIF	93.8
PIF + PVF + PMF + FIF + SIF	**99.7**

Significant values are in bold.

Table 7 presents an analysis of the classification accuracy based on different features on the Nanfang dataset. The HCNN approach obtains 56.9% classification accuracy using PIF, 55.3% classification accuracy using PVF, 59.1% classification accuracy using PMF, 63.9% classification accuracy using FIF, 65.2% classification accuracy using SIF, 70.1% classification accuracy using PIF and PVF, 68.6% classification accuracy using PIF and PMF, 75.8% classification accuracy using PIF and FIF, 94.3% classification accuracy using PIF + PVF + PMF + FIF, and 99.36% classification accuracy using PIF + PVF + PMF + FIF + SIF.

**Table 7:** Analysis of classification accuracy based on different features on Nanfang dataset.

Features	Classification accuracy (%)
PIF	**56.9**
PVF	55.3
PMF	59.1
FIF	63.9
SIF	65.2
PIF + PVF	70.1
PIF + PMF	68.6
PIF + FIF	75.8
PIF + PVF + PMF + FIF	94.3
PIF + PVF + PMF + FIF + SIF	**99.36**

Significant values are in bold.

The following Eqs. (15, 16, 17) were used to analyze the meningioma model:

15 $$Sensitivity =\frac{D}{C+D}*100\%$$

16 $$Specificity=\frac{B}{A+B}*100\%$$

17 $$Segmentation\;Accuracy=\frac{B+D}{A+B+C+D}$$

18 $$Precision \left(pr\right)=\frac{A}{A+C}*100\%$$

19 $$True\; Positive\; Rate \left(TPR\right)=\frac{A}{A+D}*100\%$$

20 $$False\; Positive \;Rate \left(TPR\right)=\frac{A}{A+D}*100\%$$

The true negative pixel pattern is represented by A, false positive pixel pattern by B, false negative pixel pattern by C, and true positive pixel pattern by D.

Table 8 presents the performance estimation of the brain tumor segmentation method using the BRATS 2019 dataset.

**Table 8:** Performance estimation of HCNN method on BRATS 2019 dataset.

BRATS image sequences	Sensitivity (%)	Specificity (%)	Segmentation accuracy (%)	Pr (%)	TPR (%)	FPR (%)
B1	99.3	99.1	99.7	99.3	98.7	99.3
B2	99.8	98.8	99.3	99.7	99.3	99.1
B3	99.2	98.9	99.1	99.2	99.1	99.8
B4	99.4	99.6	98.9	99.1	99.5	98.8
B5	99.7	99.8	98.5	99.1	99.3	98.6
B6	98.9	99.4	98.7	98.9	99.1	98.4
B7	98.6	99.7	99.3	99.3	99.2	98.7
B8	99.4	99.5	99.8	99.2	98.7	99.3
B9	99.7	99.1	99.6	99.4	98.9	99.1
B10	99.1	99.8	99.5	99.1	98.5	99.4
Average	99.31	99.37	99.24	99.23	99.03	99.05

The HCNN-based meningioma classification technique achieved 99.31% sensitivity, 99.37% specificity, and 99.24% segmentation accuracy, 99.23% of Pr, 99.03% of TPR and 99.05% of FPR, in BRATS 2019 images.

Table 9 presents the performance estimation of the brain tumor segmentation method for the Nanfang dataset.

**Table 9:** Performance estimation of HCNN method on Nanfang dataset.

Nanfang image sequences	Sensitivity (%)	Specificity (%)	Segmentation accuracy (%)	Pr (%)	TPR (%)	FPR (%)
N1	99.2	98.7	99.3	99.3	98.3	98.4
N2	99.8	98.6	98.8	99.1	98.9	99.6
N3	99.7	99.3	98.6	99.4	99.3	99.8
N4	99.3	99.1	98.1	99.1	99.1	99.4
N5	99.6	99.8	99.7	99.2	99.7	99.6
N6	99.9	99.2	99.4	98.9	99.5	99.5
N7	99.3	98.7	99.2	98.7	99.3	99.6
N8	98.7	99.7	98.9	99.3	99.1	99.4
N9	98.9	99.8	99.3	99.1	99.3	99.1
N10	99.1	99.3	99.1	99.3	99.2	99.2
Average	99.35	99.22	99.04	99.14	99.17	99.36

The proposed HCNN technique achieved 99.35% sensitivity, 99.22% specificity, and 99.04% segmentation accuracy, 99.14% of Pr, 99.17% of TPR and 99.36% of FPR on brain MRI images in the Nanfang dataset.

In this study, different segmentation methods were applied to segment the tumor pixels in the brain images, and their performances were compared in terms of classification accuracy.

Table 10 presents the simulation results of the meningioma tumor segmentation method on the BRATS 2019 dataset with respect to the different segmentation algorithms. The meningioma tumor detection method using the proposed morphological algorithm achieved a 99.31% sensitivity, 99.37% specificity, and 99.24% segmentation accuracy, 99.23% of Pr, 99.03% of TPR and 99.05% of FPR.

**Table 10:** Simulation results of the proposed HCNN method on different segmentation algorithms.

Sgmentation methods	Sensitivity (%)	Specificity (%)	Segmentation accuracy (%)	Pr (%)	TPR (%)	FPR (%)
Proposed morhological algorithm(in this work)	99.31	99.37	99.24	99.23	99.03	99.05
Existing morhological algorithm^25^	97.98	97.38	97.12	95.12	95.87	96.10
Region growing algorithm^26^	95.39	95.19	96.03	95.45	95.87	96.16

The meningioma tumor detection method using the existing morphological algorithm achieved 97.98% sensitivity, 97.38% specificity, and 97.12% segmentation accuracy.

The meningioma tumor detection method using the region-growing algorithm yielded 95.39% sensitivity, 95.19% specificity, and 96.03% segmentation accuracy.

Table 11 lists the impact of different segmentation algorithms on the Nanfang dataset with respect to. The meningioma tumor detection method using the proposed morphological algorithm achieved a 99.35% sensitivity, 99.22% specificity, and 99.04% segmentation accuracy, 99.14% of Pr, 99.17% of TPR and 99.36% of FPR.

**Table 11:** Simulation results of meningioma tumor segmentation method on Nanfang dataset with respect to different segmentation algorithms.

Sgmentation methods	Sensitivity (%)	Specificity (%)	Segmentation accuracy (%)	Pr (%)	TPR (%)	FPR (%)
Proposed morhological algorithm(in this work)	99.35	99.22	99.04	99.14	99.17	99.36
Existing morhological algorithm^25^	97.98	97.13	97.37	95.67	95.14	95.69
Region growing algorithm^26^	95.39	95.98	96.05	94.64	94.67	94.16

The meningioma tumor detection method using the existing morphological algorithm achieved 97.98% sensitivity, 97.13% specificity, and 97.37% segmentation accuracy.

The meningioma tumor detection method using the region-growing algorithm yielded 95.39% sensitivity, 95.98% specificity, and 96.05% segmentation accuracy.

In this study, different classifiers were applied to decompose brain images, and their performances were compared in terms of classification accuracy.

Table 12 shows the simulation results of the meningioma detection methods on the BRATS 2019 dataset.

**Table 12:** Simulation results of meningioma detection methods on BRATS 2019 dataset.

Methodology	Classification accuracy (%)	Sensitivity (%)	Specificity (%)	Segmentation accuracy (%)
HCNN classifier (in this work)	99.7	99.31	99.37	99.24
Existing CNN classifier^27^	98.5	98.162	98.261	98.174
Adaptive Neuro Fuzzy Inference Sustem (ANFIS) classifier^28^	97.7	95.15	95.94	95.86

**Figure 5:**
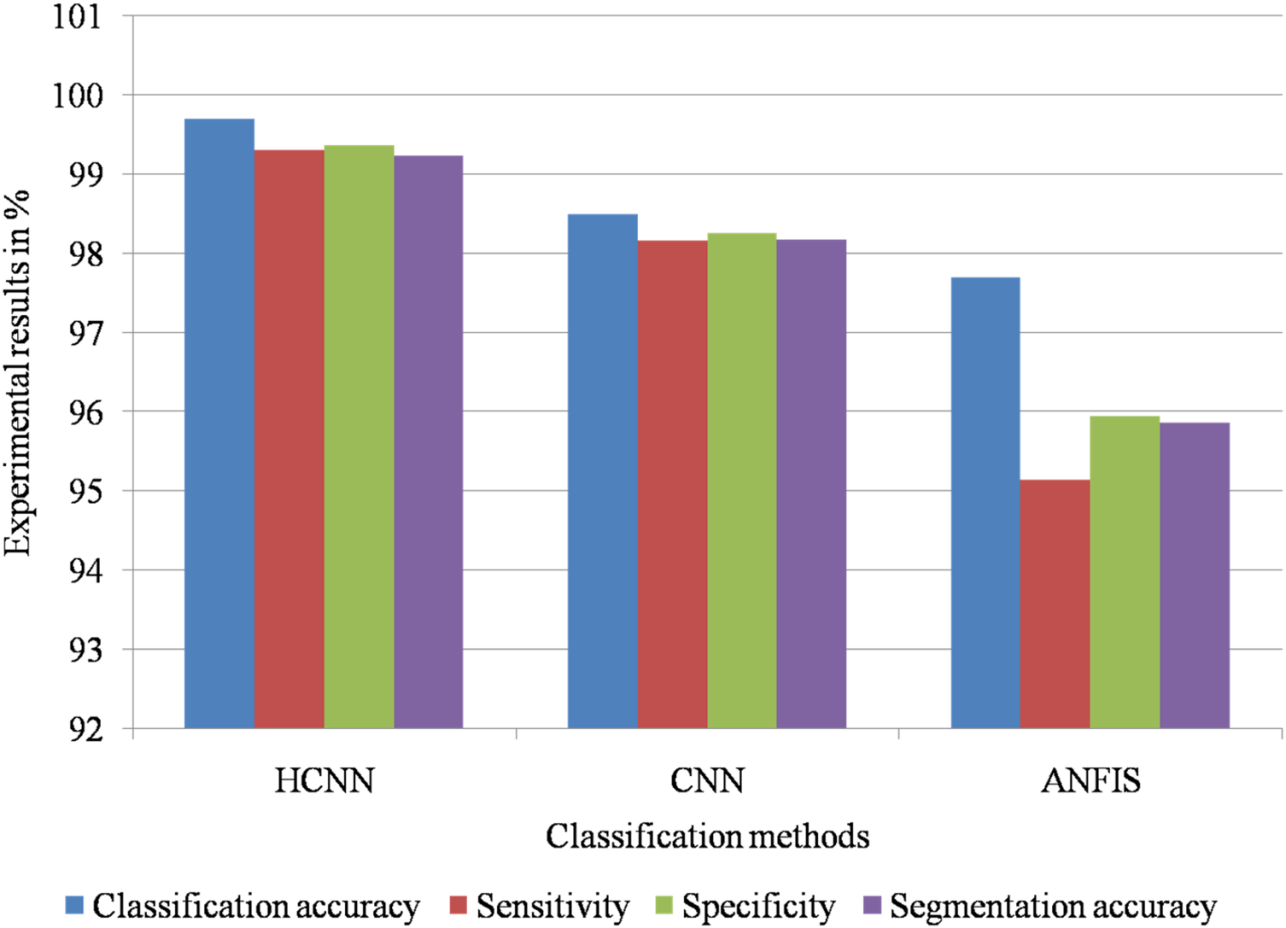
Graphical simulation results of meningioma detection methods on BRATS 2019 dataset.

Figure 5 shows the graphical simulation results of meningioma detection methods using the BRATS 2019 dataset.

Table 13 presents the simulation results of the meningioma detection methods for the Nanfang dataset.

**Table 13:** Simulation results of meningioma detection methods on Nanfang dataset.

Methodology	Classification accuracy (%)	Sensitivity (%)	Specificity (%)	Segmentation accuracy (%)
HCNN classifier (in this work)	99.36	99.35	99.22	99.04
CNN classifier^27^	98.026	98.441	98.313	98.026
ANFIS classifier^28^	95.85	96.37	96.38	96.39

**Figure 6:**
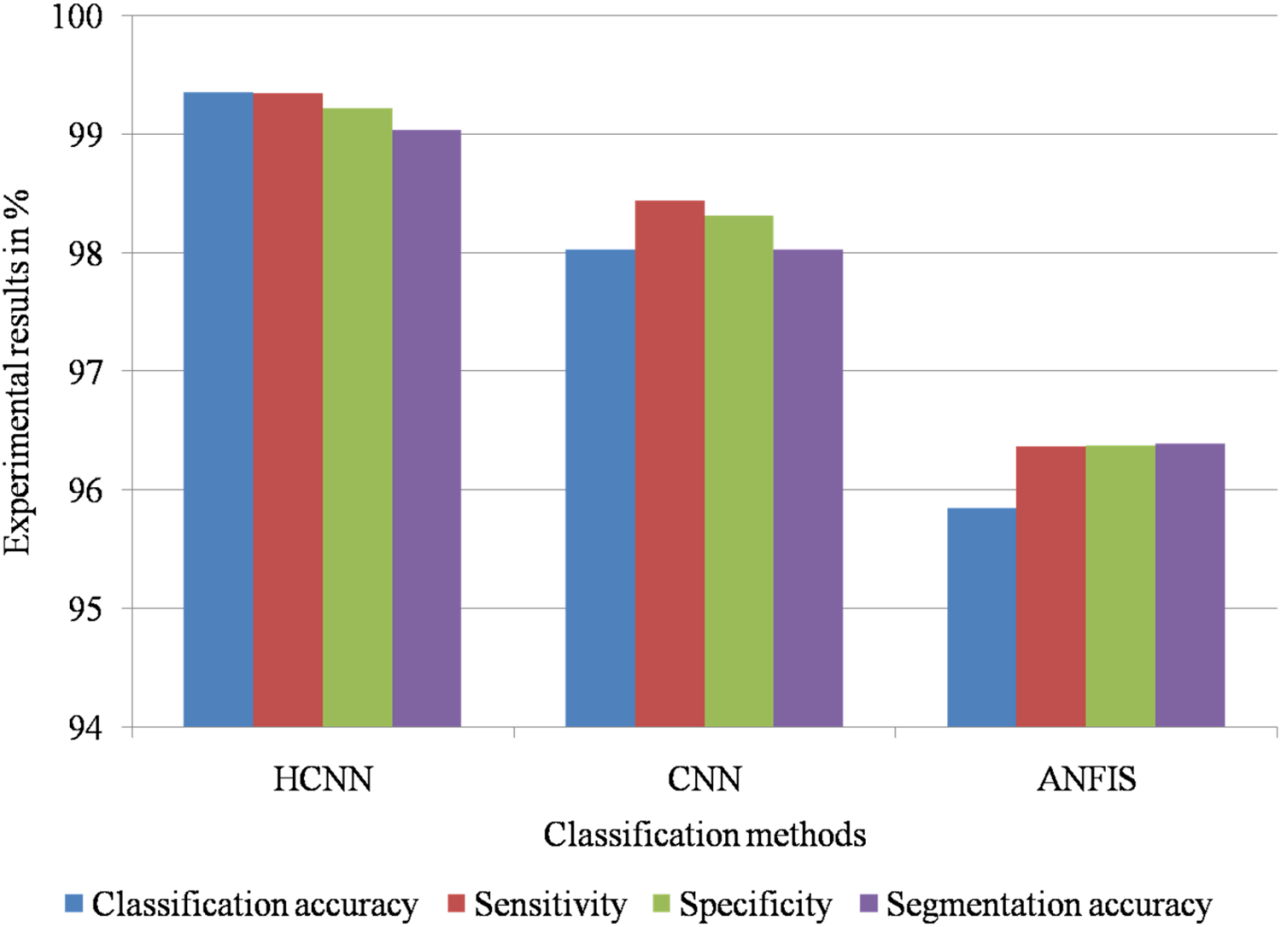
Graphical simulation results of meningioma detection methods on Nanfang dataset.

Figure 6 shows the graphical simulation results of the meningioma detection methods on the Nanfang dataset.

This meningioma detection framework was compared with conventional tumor segmentation methods, as shown in Table 13, with respect to the brain MRI images in the Nanfang dataset. As shown in Table 14, the proposed HCNN technique produces the best simulation results when compared with conventional methods^27,29–34^.

**Table 14:** Comparisons of proposed simulation results with conventional method simulation results on Nanfang dataset images.

Methodology	Classification accuracy (%)	Sensitivity (%)	Specificity (%)	Segmentation accuracy (%)
Proposed work (in this work)	99.7	99.31	99.37	99.24
^27^	**97.11**	**97.82**	**97.67**	**97.38**
^29^	96.39	96.18	96.29	96.97
^30^	97.29	97.19	97.56	97.97
^31^	96.49	96.29	97.19	96.73
^32,32^	96.96	96.39	96.85	96.39
^33^	95.18	95.85	95.38	95.21
^34^	94.85	94.28	95.10	94.49

Significant values are in bold.

This meningioma detection framework was compared with conventional tumor segmentation methods, as shown in Table 15, with respect to the brain MRI images in the BRATS 2019 dataset.

**Table 15:** Comparisons of proposed simulation results with conventional method simulation results on BRATS 2019 dataset.

Methodology	Classification accuracy (%)	Sensitivity (%)	Specificity (%)	Segmentation accuracy (%)
Proposed work (in this work)	99.36	99.35	99.22	99.04
^27^	**98.38**	**98.95**	**98.37**	**98.76**
^29^	95.98	96.12	96.38	96.87
^30^	96.19	96.57	96.28	96.86
^31^	96.38	97.16	97.97	96.75
^32,32^	97.29	97.47	97.10	97.05
^33^	95.96	96.28	95.58	95.38
^34^	94.97	94.28	94.98	94.29

Significant values are in bold.

The proposed system obtains 99.81% classification accuracy, 99.2% sensitivity, 99.7% specificity and 99.8% segmentation accuracy on BRATS 2022 dataset. Table 16 shows the comparisons of proposed simulation results with conventional method simulation results on BRATS 2022 dataset.

**Table 16:** Comparisons of proposed simulation results with conventional method simulation results on BRATS 2022 dataset.

Methodology	Classification accuracy (%)	Sensitivity (%)	Specificity (%)	Segmentation accuracy (%)
Proposed work (in this work)	99.81	99.2	99.7	99.8
^27^	**96.23**	**97.1**	**95.9**	**96.2**
^29^	95.87	96.5	94.6	96.1
^30^	95.56	95.3	94.2	95.7
^31^	94.28	95.1	93.9	94.3
^32,32^	94.09	94.9	92.8	93.2
^33^	93.28	93.2	91.8	93.9
^34^	93.68	93.1	91.0	92.9

Significant values are in bold.

## Conclusion

In this study, an HCNN classifier was proposed for the classification of brain images. The proposed HCNN technique uses the Ridgelet transform to decompose the brain image, and the pixel intensity features are then computed from the decomposed coefficients. In this study, the computed pixel intensity features were trained and classified using an HCNN classifier. The proposed HCNN-based meningioma detection system achieved 99.31% sensitivity, 99.37% specificity, and 99.24% segmentation accuracy for the BRATS 2019 dataset. The proposed HCNN technique achieved99.35% sensitivity, 99.22% specificity, and 99.04% segmentation accuracy on brain MRI images in the Nanfang dataset. The proposed system obtains 99.81% classification accuracy, 99.2% sensitivity, 99.7% specificity and 99.8% segmentation accuracy on BRATS 2022 dataset. In addition, the impact of the proposed morphological method on tumor segmentation was compared with that of other existing tumor segmentation algorithms. The major advantages of this paper are to develop an complete computer based automated method for identifying the meningioma and non-meningioma images using an efficient deep learning architecture. Moreover, the classification accuracy and performance analysis parameters are typically high using the proposed deep learning architecture when compared with existing deep learning models.

## Data Availability

The datasets used in this study were obtained from the BRATS 2019, 2022 and Nanfang datasets. This was the open-access dataset available from the following links:^1^ BRATS 2019 dataset: Accessed: Aug 10th, 2022. [Online]. https://www.kaggle.com/datasets/aryashah2k/brain-tumor-segmentation-brats-2019.^21^ Nanfang dataset: Accessed: Aug 10th, 2022. [Online]. https://www.ncbi.nlm.nih.gov/clinvar/submitters/508183/.^35^ BARTS 2022 dataset: Accessed: Aug 09th, 2023. [Online]. https://zenodo.org/record/6362180
